# Case Report Series: Aggressive HR Deficient Colorectal Cancers Related to BRCA1 Pathogenic Germline Variants

**DOI:** 10.3389/fonc.2022.835581

**Published:** 2022-02-24

**Authors:** Maria Valeria Freire, Marie Martin, Romain Thissen, Cédric Van Marcke, Karin Segers, Edith Sépulchre, Natacha Leroi, Céline Lété, Corinne Fasquelle, Jean Radermacher, Yeter Gokburun, Joelle Collignon, Anne Sacré, Claire Josse, Leonor Palmeira, Vincent Bours

**Affiliations:** ^1^Department of Human Genetics, GIGA Research Center – University of Liège and Centre Hospitalier Universitaire (CHU) Liège, Liège, Belgium; ^2^Institute for Experimental and Clinical Research (Institut de Recherche Expérimentale et Clinique (IREC), Pôle Molecular Imaging, Radiotherapy and Oncology (MIRO)), Université Catholique de Louvain (UCLouvain), Brussels, Belgium; ^3^Department of Medical Oncology, Institut Roi Albert II, Cliniques Universitaires Saint‐Luc, Brussels, Belgium; ^4^Department of Pathology, Institut de Pathologie et de Génétique, Charleroi, Belgium; ^5^Department of Gastroenterology, Centre Hospitalier Régional Sambre et Meuse, Namur, Belgium; ^6^Department of Medical Oncology, GIGA Research Center – University of Liège and Centre Hospitalier Universitaire (CHU) Liège, Liège, Belgium; ^7^Onco-Hematology Department, Centre Hospitalier Régional (CHR) Verviers, Verviers, Belgium

**Keywords:** colorectal (colon) cancer, *BRCA1*, homologous recombination deficiency (HRD), exome sequencing (ES), case report

## Abstract

**Objective:**

The link between *BRCA1* and homologous recombination deficiency (HRD) in cancer has gained importance with the emergence of new targeted cancer treatments, while the available data on the role of the gene in colorectal cancer (CRC) remain contradictory. The aim of this case series was to elucidate the role of known pathogenic *BRCA1* variants in the development of early-onset CRC.

**Design:**

Patients were evaluated using targeted next generation sequencing, exome sequencing and chromosomal microarray analysis of the paired germline and tumor samples. These results were used to calculate the HRD score and the frequency of mutational signatures in the tumors.

**Results:**

Three patients with metastatic CRC were heterozygous for a previously known *BRCA1* nonsense variant. All tumors showed remarkably high HRD scores, and the HRD-related signature 3 had the second highest contribution to the somatic pattern of variant accumulation in the samples (23% in 1 and 2, and 13% in sample 3).

**Conclusions:**

A *BRCA1* germline pathogenic variant can be involved in CRC development through HRD. Thus, *BRCA1* testing should be considered in young patients with a personal history of microsatellite stable CRC as this could further allow a personalized treatment approach.

## Introduction

*BRCA1* is a tumor suppressor gene encoding a large protein that coordinates several cellular pathways including DNA repair, transcriptional regulation, cell-cycle control, centrosome duplication, and apoptosis (1). Pathogenic germline variants in *BRCA1* gene have been associated with familial risk of breast and ovarian cancers (OMIM: 604370) (2, 3). As early as in 1994, it was observed that women with a history of breast, endometrial, or ovarian cancer presented a statistically significant although small risk for subsequent colorectal cancer (CRC), suggesting the existence of common etiologic factors for the development of these tumors (4).

Data concerning young patients with *BRCA1* variants that develop CRC have been scarce. Germline pathogenic variants in *BRCA1* gene have not been causally linked to an increased risk of familial colorectal cancer, but the reports on the subject are contradictory (5–9). Indeed, patients carrying a germline *BRCA1* variant can develop a sporadic tumor, independently of *BRCA1* loss of function, highlighting the need to demonstrate the causal role of the variant in the cancer development (10).

The aim of this case series was to gain insight into the role of known pathogenic *BRCA1* variants in the development of early-onset CRC.

## Case Description

Three patients were diagnosed in 2020 and 2021 with aggressive early-onset CRC. The demographic, familial, clinical, histopathological, and molecular characteristics, as well as the treatment regimens of these patients are presented in **Table 1**.

**Table 1:** Patient characteristics.

Parameter	Case 1	Case 2	Case 3
Age (year)	31	56	35
Sex	Female	Female	Male
Medical history	None	Breast cancer at 36 y/o, contralateral breast cancer at 41 y/o	Ulcerative colitis
Family history	Maternal side: aunt breast cancer, grandmother CRC, great-grandmother uterine cancer	Paternal side: aunt CRC, grandmother ovary cancer, grandmother’s sister breast cancer	Maternal side: five aunts breast cancer, grandmother ovary cancer.
			Paternal side: grandmother CRC
CRC localization and type	Right colon moderately differentiated adenocarcinoma	Well to moderately differentiated rectum adenocarcinoma	Mucinous appendix adenocarcinoma
TNM tumor staging	pT4aN2aM1a	cT3N1M1b	pT4bN0M0 at diagnosis, peritoneal relapse at month 5
**IHC and molecular tests on the tumor**			
MSI-H	No	No	No
MLH1, MSH2, MSH6 and PMS2 protein expression	Normal	Normal	Normal
**Identified variants**			
Somatic pathogenic variants (heterozygous)	*KRAS* c.35G>A (p. Gly12Asp)	*KRAS* c.35G>A (p. Gly12Asp)	–
	*TP53* c.524G>A (p.Arg175His)		
Germline pathogenic variants (heterozygous)	*BRCA1* c.1016dup (p.Val340Glyfs*6)	*BRCA1* c.3756_3759del (p.Ser1253Argfs*10)	*BRCA1* c.3841C>T (p.Gln1281*)
**Somatic CMA array results**	Partial gains and losses on Chr 1-3, 5- 9, 12, 13, 15-20 and X	Entire and partial gains and losses on Chr 1, 7, 8, 12, 13 and 18-20	Normal
**HRD evaluation**			
HRD score	59	61.15	66
Proportions of mutational signatures with a proposed etiology			
SBS1	24%	28%	14%
SBS3	23%	22%	13%
SBS5	0%	0%	20%
**Treatment**			
Surgical	Right colectomy with lymph node dissection, ileocolonic anastomosis and metastasectomy of liver segments	Anterior rectum resection and hepatic surgery	Ileocolectomy with a lymph node dissection firstly and a posterior debulking surgery with IPCH after discovery of a peritoneal carcinomatosis
Chemotherapy	Pseudo-adjuvant chemotherapy with capecitabine-oxaliplatin followed by 7 cycles of chemotherapy with FOLFOX-bevacizumab	6 cycles of FOLFOXIRI	Adjuvant chemotherapy with capecitabine-oxaliplatin regimen (Xelox)

CMA, chromosomal microarray analysis; FOLFOX, folinic acid; fluorouracil and oxaliplatin; FOLFOXIRI, fluorouracil; folinic acid; oxaliplatin; and irinotecan; HRD, homologous recombination deficiency; IHC, Immunohistochemistry; IPCH, Intraperitoneal chemohyperthermia; MMR, mismatch repair; MSI, microsatellite instability; SBS, Single Base Substitution; TNM, TNM Classification of Malignant Tumors; y/o, years old. Reference transcripts: BRCA1 NM_007294.3; KRAS NM_004985.5; TP53 NM_000546.6.

The first case was referred to oncogenetic consultation due to the young age of presentation of an aggressive disease without evidence of Lynch syndrome (no mismatch repair deficiency or microsatellite instability) and history of a *BRCA1* pathogenic variant in the family. Given the age at the diagnosis of CRC, genes associated with familial polyposis (*NTHL1, RNF43, SMAD4, BMPR1A*), CRC (*POLE, POLD1*) and Li-Fraumeni syndrome (*TP53*) were analyzed. However, the patient only carried the heterozygous *BRCA1* pathogenic variant NM_007294.3(BRCA1_v001):c.1016dup (p.Val340Glyfs*6) identified in her maternal aunt.

To further evaluate the disease, targeted next-generation sequencing (NGS) and a high-resolution (180K) chromosomal microarray analysis (CMA) were performed on the DNA extracted from the tumor (estimated proportion of tumor cells in the sample - 50%). After sequencing, the familial pathogenic variant *BRCA1* c.1016dup was identified at an allele frequency (AF) of 70%, suggesting a loss of heterozygosity at the *BRCA1* locus. Further analysis revealed a somatic variant of *TP53* NM_000546.6(TP53):c.524G>A (p.Arg175His) at an AF of 40%. The CMA showed multiple rearrangements indicating genomic instability (chromosomal partial gains and losses on chromosomes 1-3, 5- 9, 12, 13, 15-20 and X).

The personal and family history of cancer in case 2 already led in 2011 to the identification of the pathogenic *BRCA1* germline variant NM_007294.3(BRCA1_v001):c.3756_3759del (p.Ser1253Argfs*10). Taking this information into account, a CMA and NGS of the tumor DNA (estimated tumor infiltration – 30%) were performed, identifying the known germline *BRCA1* variant with an AF of 35% and an additional NM_004985.5(KRAS_v001):c.35G>A variant with an AF of 23%. The CMA results were monosomies 18 and 19, trisomies 1q, 7, 8, 12, 13 and 20, partial chromosomal losses in the 1p region and partial chromosomal gains in the 1p region.

In case 3, CRC was diagnosed from a surgical specimen obtained after an appendectomy with the subsequent identification of a tumor-like lesion with low-grade dysplasia at the base of the cecum. Considering that the patient’s mother carried a *BRCA1* germline variant, the patient DNA was tested, confirming the presence of the heterozygous BRCA1 pathogenic variant NM_007294.3(BRCA1_v001):c.3841C>T (p.Gln1281*). Subsequently, *BRCA1* sequencing and CMA array on tumor DNA (sample estimated tumor infiltration – 20%) showed the *BRCA1* c.3841C>T family variant with an AF of 43%, while the CMA was normal.

The three variants are predicted to cause truncation of the translation in exon 10 (out of a total of 23) which will result in a severely shortened or absent protein due to nonsense-mediated decay of the mRNA. *BRCA1* protein truncations downstream of this position have been described as pathogenic (11, 12). *BRCA1* c.1016dup and *BRCA1* c.3841C>T variants were absent in 251174 control chromosomes in gnomAD, whereas *BRCA1* c.3756_3759del was present at an AF of 1.267e-05. *BRCA1* c.1016dupA has been reported in the literature as a founder variant in Norway and Canada (13, 14) and also in multiple individuals affected with hereditary breast and ovarian cancer syndrome in other populations (15–18). Case 2 four-nucleotide deletion was widely reported in the literature in Polish and French-Canadian gynecological cancer patients (19, 20). The *BRCA1* variant present in case 3 has been reported as a France, Belgium, and Holland founder variant (21). ClinVar submitters including an expert panel (ENIGMA) cite the three variants as pathogenic. These data indicate that the three variants are highly likely to be associated with high breast and ovarian cancer risk.

Homologous recombination deficiency (HRD) evaluation can be performed using HRD score, an aggregate score of loss of heterozygosity (LOH), telomeric-allelic imbalance (TAI) and large-scale state transitions (LST). To confirm the HRD score in the CRC samples we used an alternative method of HRD detection by investigating single base substitution (SBS) signatures.

To assess homologous recombination deficiency (HRD) in CRC samples, a paired germline and tumoral DNA exome sequencing using Twist Comprehensive Exome Panel and Twist Human RefSeq Panel (according to the manufacturer’s instructions) from all three patients was performed. We used Sequenza (22) to detect and quantify copy number variation and estimate tumor cellularity and ploidy. These results were used as an input to calculate the HRD score with a threshold of positivity ≥33 (23). Mutational signatures in the samples were analyzed Using MutationalPatterns R package (24) and COSMIC v2 signatures (25), taking only the somatic variants into account.

Through Sequenza, the estimated tumor cellularity was of 95% in the case 3 sample, while this value was lower for cases 1 and 2 – 22% and 27%, respectively. All three samples showed remarkably high HRD scores (59, 61.15 and 66, respectively), while no somatic copy number alteration was identified in *PALB2, BRCA1* and *BRCA2*.

**Figure 1:**
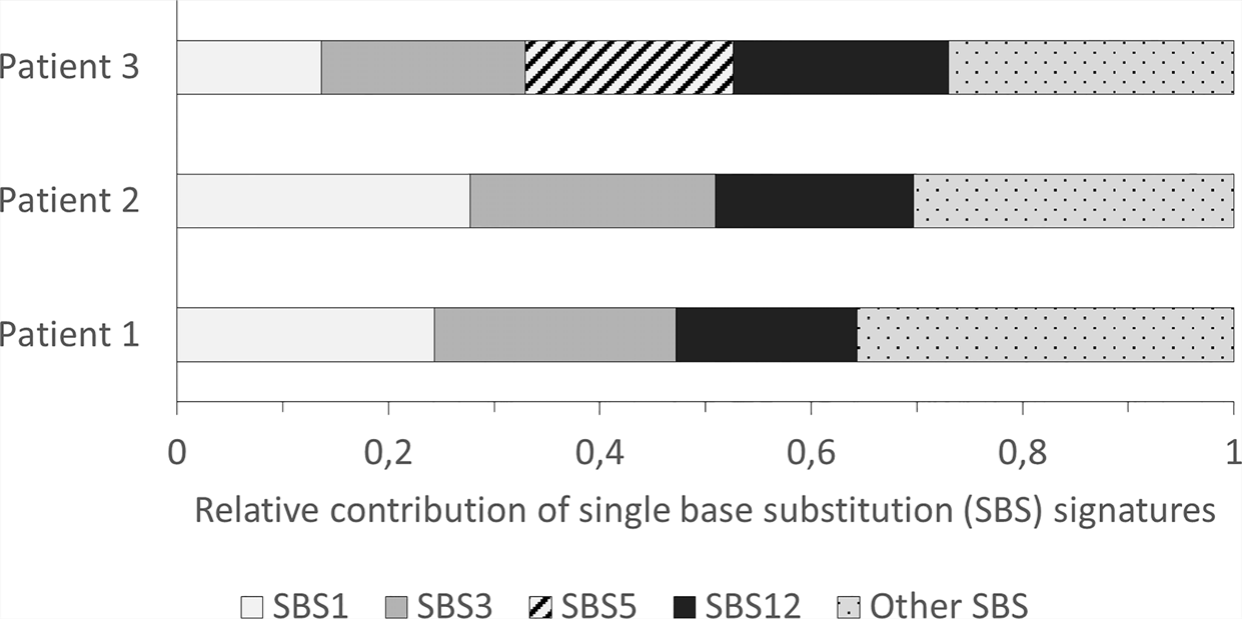
Single base substitution (SBS) signatures identified in the patients with a relative contribution ≥ 15% of the global pattern.

The three most frequent SBS signatures with a proposed etiology in the samples were SBS1, 3 and 5 (see **Figure 1**). Signature 3 was the second most frequent signature with a contribution to 23% of the somatic pattern of variant accumulation in samples 1 and 2, and 13% in sample 3. While signature 1 and 5 reflect clock-like accumulation of somatic variants, signature 3 has been directly related to HRD (25).

## Discussion

The existing data linking germline pathogenic variants in the *BRCA1* gene to an increased risk of CRC are scarce. Two large studies reported that *BRCA1* variants conferred approximatively a fivefold increased risk for CRC, especially in young patients from high-risk families (6, 26). Out of three recent meta-analyses, one of them found an increased risk of colorectal cancer associated with *BRCA1* variants (odds ratio = 1.49, 95% CI = 1.19 to 1.85, P < 0.001) (8), while the other two did not identify any increase in CRC risk among patients carrying a *BRCA1* variant (7, 9). A study evaluating a cohort of *BRCA1* or *BRCA2* pathogenic variant carriers mostly of Ashkenazy ancestry concluded that they may be prone to developing anal carcinoma and left-sided mucinous histology CRC (27). One single publication reported a young male patient with a *BRCA1* germinal variant who presented with rectal adenocarcinoma and showed an excellent response to oxaliplatin-containing neoadjuvant therapy (28). These data thus remain contradictory and do not allow to recommend to screen for CRC in BRCA1 variants heterozygotes, or to consider *BRCA1* pathogenic variants as a factor predisposing to familial CRC.

Given the frequency of CRC and of *BRCA1* variant heterozygotes in European populations (29), co-occurrence may be incidental rather than indicative of a causal relationship, as suggested previously (30). However, a few lines of evidence indicate that co-occurrence might be relevant.

Recently, a large report investigated the frequencies of various cancers, including CRCs, in 6902 men with BRCA variants (31). The probability for developing a CRC was, according to this report, two times lower in men with *BRCA2* variants than in *BRCA1* variant heterozygotes. As it seems unlikely that *BRCA2* variants had a protective role against CRCs, these data could indicate a slightly but significantly increased risk of these cancers in men with *BRCA1* variants.

In our samples, we did not evaluate BRCA1 protein expression. Although we describe patients with aggressive metastatic cancer, the presence of low levels of BRCA1 protein had a worse prognosis even in early-stage CRC (32).

In our study, we not only confirmed that the *BRCA1* germline variants were still present in the tumor (with evidence of positive selection in case 1), but we also demonstrate scars of HRD in the three tumors. Indeed, the presence of germline variants in HRD-associated genes alone is not sufficient to predict clinically relevant HRD. We highlighted the presence of specific mutational signatures (COSMIC signature 3) (33) and genomic instability characteristics (LOH, TAI and LST) (34–36), reflecting significant HRD, comparable with that observed in ovarian cancers with a *BRCA1* or *BRCA2* pathogenic variants. Interestingly, the initial somatic NGS analysis of cases 2 and 3 was not conclusive, possibly because of low tumor infiltration, but it could also be indicative of an epigenetic event leading to loss of *BRCA1* function and demonstrates the role of HRD testing even in cases where the mechanism driving HRD is not fully elucidated. Taken together, these observations indicate that germline *BRCA1* variants may, in a small proportion of variant carriers, play a driver role in CRC development or progression and that these patients might thus benefit from a treatment with poly (ADP-ribose) polymerase-inhibitors (PARPi). Indeed, clinical trials clearly demonstrated the efficacy of platinum-based chemotherapy and PARPi to treat BRCA mutated and/or HRD positive cancers inside the spectrum of BRCA-related cancers (37). Further evidence demonstrating that some CRC could be linked to BRCA deficiencies could open new perspectives for treatment with PARPi of these rare aggressive tumors.

The small number of patients and the bias in recruitment are the main limitations of our study, precluding to justify any specific surveillance or screening program in the absence of a personal or family history.

In conclusion, our data indicate that a *BRCA1* germline pathogenic variant can be involved in CRC development through HRD. Thus, *BRCA1* testing should be considered in young patients with a personal history of microsatellite stable CRC. This could further allow a personalized treatment approach with a PARPi.

## Data Availability Statement

The original contributions presented in the study are included in the article/supplementary material. Further inquiries can be directed to the corresponding author.

## Ethics Statement

The “Comité d’Ethique Hospitalo-facultaire Universitaire de Liège” (CHU/University of Liège) approved the study. The patients/participants provided their written informed consent to participate in this study. Written informed consent was obtained from the individual(s) for the publication of any potentially identifiable images or data included in this article.

## Author Contributions

Conceptualization: VB. Data curation: MF, MM, and VB. Formal analysis: MF. Funding acquisition: VB. Investigation: MF, MM, RT, CM, KS, ES, NL, CL, and CF. Methodology: MF, MM, RT, CM, CJ, and LP. Project administration: VB. Resources: VB. Supervision: CJ, LP, and VB. Validation: JR, YG, JC, and AS. Writing-original draft: MF and VB. Writing-review and editing: MF, MM, RT, CM, KS, ES, NL, CL, JR, YG, JC, AS, CJ, LP, VB, and CF. All authors contributed to the article and approved the submitted version.

## Funding

This work was supported by a Télévie fellowship (MV.F., grant N° 7451419F), a grant from the CHU Liége (N° 981481200) and the WALGEMED grant (Région Wallonne, grant N° 1710180).

## Conflict of Interest

The authors declare that the research was conducted in the absence of any commercial or financial relationships that could be construed as a potential conflict of interest.

## Publisher’s Note

All claims expressed in this article are solely those of the authors and do not necessarily represent those of their affiliated organizations, or those of the publisher, the editors and the reviewers. Any product that may be evaluated in this article, or claim that may be made by its manufacturer, is not guaranteed or endorsed by the publisher.
